# VA FitHeart, a Mobile App for Cardiac Rehabilitation: Usability Study

**DOI:** 10.2196/humanfactors.8017

**Published:** 2018-01-15

**Authors:** Alexis L Beatty, Sara L Magnusson, John C Fortney, George G Sayre, Mary A Whooley

**Affiliations:** ^1^ Veterans Affairs Puget Sound Health Care System Seattle, WA United States; ^2^ Department of Medicine University of Washington Seattle, WA United States; ^3^ Department of Psychiatry University of Washington Seattle, WA United States; ^4^ San Francisco Veterans Affairs Medical Center San Francisco, CA United States; ^5^ Department of Medicine University of California, San Francisco San Francisco, CA United States; ^6^ Department of Epidemiology and Biostatistics University of California, San Francisco San Francisco, CA United States

**Keywords:** cardiac rehabilitation, mobile applications, exercise therapy, exercise, rehabilitation research, telemedicine, habits, qualitative research

## Abstract

**Background:**

Cardiac rehabilitation (CR) improves outcomes for patients with ischemic heart disease or heart failure but is underused. New strategies to improve access to and engagement in CR are needed. There is considerable interest in technology-facilitated home CR. However, little is known about patient acceptance and use of mobile technology for CR.

**Objective:**

The aim of this study was to develop a mobile app for technology-facilitated home CR and seek to determine its usability.

**Methods:**

We recruited patients eligible for CR who had access to a mobile phone, tablet, or computer with Internet access. The mobile app includes physical activity goal setting, logs for tracking physical activity and health metrics (eg, weight, blood pressure, and mood), health education, reminders, and feedback. Study staff demonstrated the mobile app to participants in person and then observed participants completing prespecified tasks with the mobile app. Participants completed the System Usability Scale (SUS, 0-100), rated likelihood to use the mobile app (0-100), questionnaires on mobile app use, and participated in a semistructured interview. The Unified Theory of Acceptance and Use of Technology and the Theory of Planned Behavior informed the analysis. On the basis of participant feedback, we made iterative revisions to the mobile app between users.

**Results:**

We conducted usability testing in 13 participants. The first version of the mobile app was used by the first 5 participants, and revised versions were used by the final 8 participants. From the first version to revised versions, task completion success rate improved from 44% (11/25 tasks) to 78% (31/40 tasks; *P*=.05), SUS improved from 54 to 76 (*P*=.04; scale 0-100, with 100 being the best usability), and self-reported likelihood of use remained high at 76 and 87 (*P*=.30; scale 0-100, with 100 being the highest likelihood). In interviews, patients expressed interest in tracking health measures (“I think it’ll be good to track my exercise and to see what I’m doing”), a desire for introductory training (“Initially, training with a technical person, instead of me relying on myself”), and an expectation for sharing data with providers (“It would also be helpful to share with my doctor, it just being a matter of clicking a button and sharing it with my doctor”).

**Conclusions:**

With participant feedback and iterative revisions, we significantly improved the usability of a mobile app for CR. Patient expectations for using a mobile app for CR include tracking health metrics, introductory training, and sharing data with providers. Iterative mixed-method evaluation may be useful for improving the usability of health technology.

## Introduction

Cardiac rehabilitation (CR) is an evidence-based program of exercise training, risk factor management, education, and counseling that improves outcomes for patients with heart disease [1-4]. However, CR is dramatically underused, with less than 20% of eligible patients participating [5-7] *.* Many barriers limit participation, including expectations for attending facility-based supervised exercise sessions three times per week for 12 weeks, transportation difficulties, competing demands related to work or family, lack of social support, and cost [8-10]. Home-based CR programs are similar in efficacy and safety to facility-based programs but have not been widely adopted in the United States [11,12]. New strategies are needed to promote participation in home-based CR [13,14].

Technology has the potential to facilitate health interventions and motivate patients to improve health behaviors, including for the secondary prevention of cardiovascular disease [15-19]. It is known that interventions with a theoretical basis are more effective [20], but most technology solutions have not been created around evidence-based practices or health behavior theory [18,21-23]. The Theory of Planned Behavior (TPB) [24] has been successfully applied to CR in both facility- and home-based settings [25,26]. The TPB states that the most important determinant of behavior is the intention to perform the behavior. Behavioral intention is influenced by constructs of attitudes, subjective norms, and perceived behavioral control. An extension of the TPB has been developed to explain behavior specific to technology use, called the Unified Theory of Acceptance and Use of Technology (UTAUT) [27] and its extension for consumer use of technology (UTAUT2) [28]. This theory contends that constructs of performance expectancy, effort expectancy, social influence, facilitating conditions, hedonic motivation, price value, and habit influence behavioral intention, which is the strongest predictor of technology use.

Using the TPB and UTAUT2, we developed a theory-based mobile app for technology-facilitated home CR. We tested the mobile app in patients eligible for CR, obtained feedback, and iteratively made revisions to the mobile app to improve its usability. Additionally, we interviewed participants about physical activity, CR, and mobile app use to better understand how to implement technology-facilitated home CR. The aims of this study were to determine the usability of the VA FitHeart mobile app and to analyze factors contributing to its use.

## Methods

### Overview

**Figure 1:**
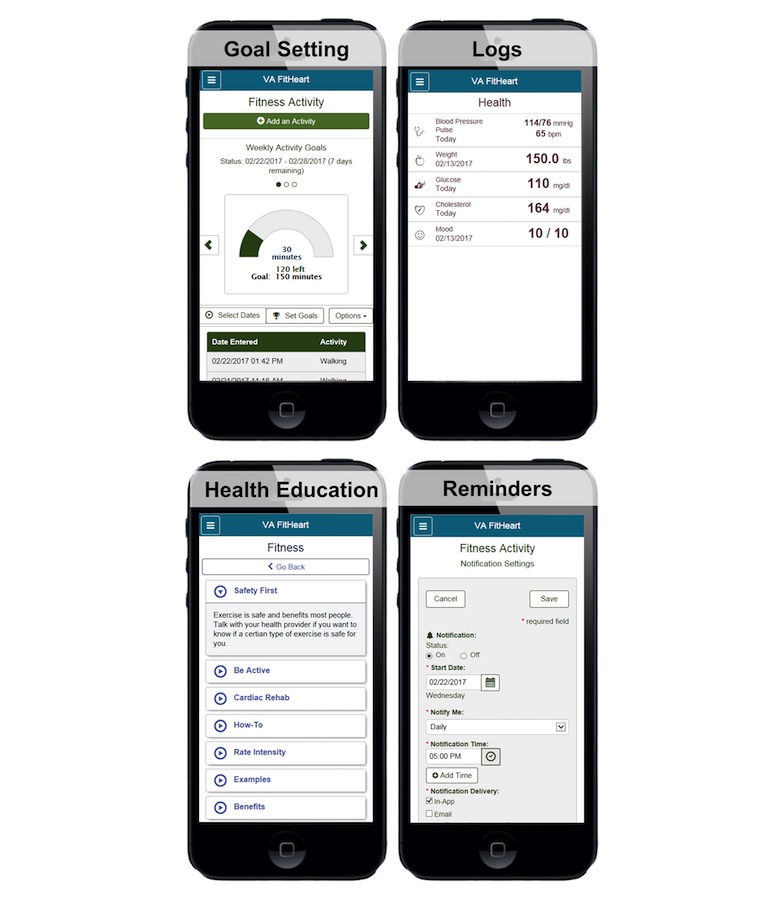
Screenshots of VA FitHeart, a mobile app for cardiac rehabilitation.

We conducted an observational study of Veteran use of a mobile Web app, VA FitHeart. The mobile app was designed to be used as a tool for home CR and includes physical activity goal setting, logs for physical activity and health measures (eg, blood pressure, pulse, weight, glucose, cholesterol, and mood), health education, reminders, and feedback (Figure 1). The mobile app was developed by the Department of Veterans Affairs (VA), and testing was conducted on versions of the mobile app hosted on preproduction testing servers. VA FitHeart was designed with input from subject matter experts (including the authors), patients eligible for CR, user experience designers, and mobile app developers. VA FitHeart underwent iterative revision based on review from VA mobile compliance bodies, including human factors, section 508 compliance, patient safety, data and terminology standardization, branding, and data security. During the course of this study, VA FitHeart underwent iterative user interface revisions based on participant feedback on usability. Because the app was hosted in a testing environment, there were occasional server downtimes when the app was not accessible for testing.

### Participants

Veterans attending the outpatient cardiology clinic at the VA Puget Sound Health Care System in Seattle, WA were screened for enrollment in the study. Eligibility criteria included the ability to speak English, age ≥21, and eligibility for CR, defined as myocardial infarction, percutaneous coronary intervention, or cardiac surgery in the past year or having chronic stable angina or heart failure. Participants were excluded if they were not eligible for CR. Participants meeting inclusion criteria were asked to participate in additional screening to participate in a study about a mobile app for CR. Participants were excluded if they did not have access to a mobile phone, tablet, or computer with Internet access. This study was reviewed and approved by the institutional review board at the VA Puget Sound Health Care System. All participants provided written, informed consent.

### Usability Testing

Study staff demonstrated the mobile app to participants in person and asked participants to complete prespecified tasks with the mobile app while study staff observed the participants. Tasks demonstrated by study staff included setting a physical activity goal, making a physical activity entry, viewing a fitness graph, making a weight entry, and viewing an educational module. After the conclusion of the demonstration, participants were asked to complete the demonstrated tasks independently. Study staff recorded task completion success if the participant was able to successfully complete the task.

### Questionnaires

Following testing, participants completed questionnaires using REDCap electronic data capture tools hosted at the VA [29], including rating their likelihood to use the mobile app from 0 (low) to 100 (high). Participants completed the System Usability Scale (SUS) [30], with scoring from 0 to 100, with ratings of greater than 70 generally considered to demonstrate acceptable usability [31]. In addition, participants rated factors influencing mobile app use related to constructs from UTAUT2, including performance expectancy, social influence, facilitating conditions, habit, hedonic motivation, price value, and behavioral intention (scale 0-100; Multimedia Appendix 1) [28]. Effort expectancy was operationalized as response to the SUS.

### Interviews

We conducted two separate semistructured interviews with Veterans enrolled in the study. The first interview was conducted before usability testing and was centered on physical activity and the use of technology. The second interview was conducted before usability testing, asking specific questions about the functionality of the mobile app. All interviews took place in person at the VA Puget Sound Health Care System in Seattle, WA in a private office. Both interviews had semistructured interview guides that included open-ended questions and prompts for elicitation of additional detail (Multimedia Appendix 2). Interviews were conducted by two trained study staff members, audiorecorded, and transcribed word for word.

### Analysis

Descriptive statistics of range and mean were used for quantitative questionnaire responses. To compare responses before and after, we performed a two-tailed *t* test. Qualitative interviews were transcribed and coded using Atlas.ti (Atlas.ti GmbH) version 7.2. Two researchers (ALB and SLM) coded interviews; we performed inductive and deductive content analysis [32]. We used a priori categories from the constructs of the UTAUT for consumer applications (performance expectancy, effort expectancy, social influence, facilitating conditions, habit, hedonic motivation, and price value) and the TPB (attitudes, subjective norms, and perceived behavioral control). In addition, we generated additional codes that emerged naturally based on participant responses. Both researchers wrote analytic memos to document observations and participated in intermittent meetings to discuss emergent themes, add or collapse codes, and reach consensus on coding disagreements. The research team conducted a thematic analysis to assess patterns of experiences and opinions across themes and reached agreement of interpretation. Analysis was conducted concurrently with participant enrollment. We continued enrollment of new participants until we had achieved acceptable usability and stakeholders believed that sufficient data had been collected to make the decision to not make additional revisions. The results of the study are reported in accordance with the Consolidated Standards of Reporting Trials of Electronic and Mobile HEalth Applications and onLine TeleHealth checklist [33].

## Results

### Participant Characteristics

From January 27, 2016 to October 24, 2016, we enrolled 15 participants in usability testing (Multimedia Appendix 3). Participants ranged in age from 43 to 75 years (mean 63 years). There were 14 males and 1 female, and 13 participants identified race as white (87%). Primary diagnoses included coronary artery bypass surgery (2/15, 13%), percutaneous coronary intervention (3/15, 20%), chronic stable angina (5/15, 33%), and stable heart failure (6/15, 40%).

### Usability Testing

**Figure 2:**
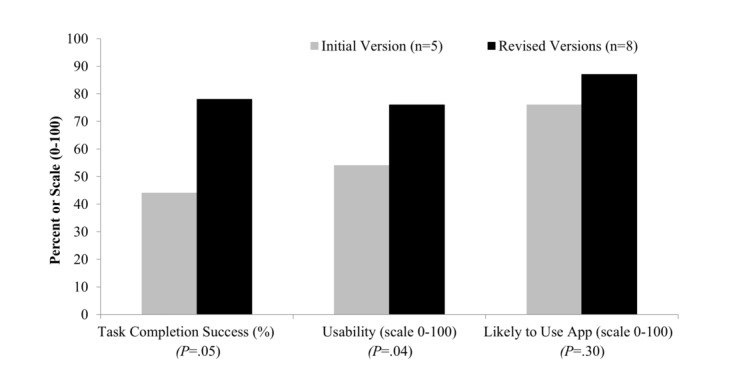
Task completion success and patient-reported usability and likelihood of using the mobile app on initial and revised versions of the mobile app. Task completion success was the percentage of tasks successfully completed. Usability was score on the System Usability Scale (scale 0-100, with 100 being the best usability). Likely to use app was self-rated likelihood of use (scale 0-100, with 100 being the highest likelihood). *P* values represent comparisons between the initial version and revised versions.

**Figure 3:**
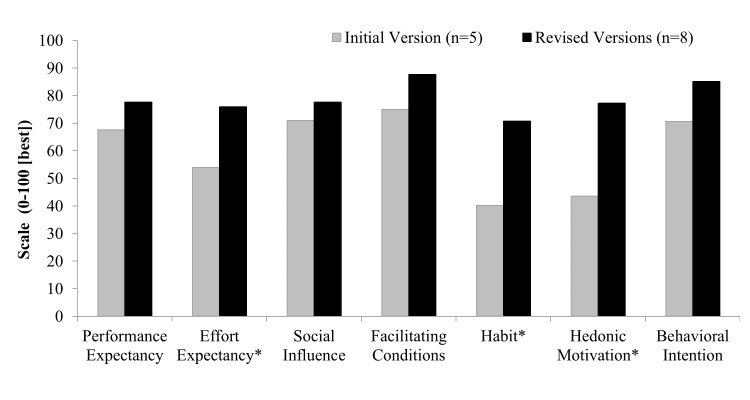
Patient-reported factors influencing mobile app use on initial and revised versions of the mobile app. Items were rated on a scale of 0 to 100, with 100 being the best rating. *P<.05 for comparison between versions.

The first version of the mobile app was used by the first 5 participants, and revised versions were used by 8 participants. Two participants were unable to complete testing because of technical difficulties with accessing the servers in the preproduction testing environment during server downtimes. From the first version to revised versions, task completion success rate improved from 44% (11/25 tasks) to 78% (31/40 tasks; *P*=.05), SUS improved from 54 to 76 (*P*=.04; scale 0 to 100, with 100 being the best usability), and rated likelihood of using the mobile app remained high at 76 and 87 (*P*=.30; scale 0 to 100, with 100 being the highest likelihood; Figure 2). We found that revised versions of the mobile app significantly improved constructs from UTAUT2, including effort expectancy, habit, and hedonic motivation (Figure 3).

### Mobile Technology Use

Emergent themes about mobile technology use were categorized by UTAUT2 construct (Multimedia Appendix 4).

### Performance Expectancy

Many participants expect that VA FitHeart would be beneficial.

I think that the idea of an app that records all of the information that this app is doing will be very valuable. Actually somewhat of a motivation for me to do this thing.

P28

Participants desired that a mobile app for CR be able to track goals, physical activity, and other health measures such as blood pressure, heart rate, weight, blood glucose, and diet.

Although there were suggestions for additional features to the mobile app, such as the ability to integrate with sensors and automatically transfer data, it was commented that this was not essential.

Memorizing, writing it down and then getting it into your computer, if that was all done while you’re doing activities and stuff that would be a big help. But if they can’t, this is still a good app. Still helpful.

P28

### Effort Expectancy

Several aspects of ease of use of the mobile app emerged. Participants appreciated simplicity.

It was pretty easy...I like that it’s simple.

P45

The flow is very simple.

P07

Vision and size of text were cited as a barrier by many participants.

The only downside I see for me is with my vision; the fonts are a little small. I would definitely need to use my reading glasses to read it.

P44

Prominent display of key features was cited as a facilitator of ease of use.

The settings to change your goals are very easy to reach and very prominent.

P23

Although some users commented on functions that were not as intuitive and harder to find, it was recognized that with more experience and familiarity, this problem could be overcome.

I’m not used to this. Once I get used to it, I’ll know where everything is.

P40

One general barrier to ease of use mentioned by participants was the use of passwords and codes. This did not emerge as a barrier specific to our app, but participants were not required to enter a password during the testing session.

### Social Influence

Participants often mentioned a desire to share their data with their providers.

I like the fact that I can put all of that and track it, and that my doctors can as well. I can show my doctor what I’ve been working on.

P45

There was also interest in communicating with providers through the app. Family and peer support were reported to influence mobile technology use. The mobile app does feature a link to an online social networking site for patients with heart disease, but social networking was infrequently mentioned.

### Facilitating Conditions

A desire for hands-on initial training on how to use the mobile app emerged as an important theme.

Initially, training with a technical person, instead of me relying on myself.

P8

Expectations for additional help varied, including online, telephone, and family or peer support.

If I had problems I’d try to find out how to fix it on this or call you.

P40

But I’ve got 3 boys that are all pretty much wizards at it, but I’m not. I’m sure I can learn it or if they punch in the application so that it could come right up, I’d be fine.

P19

### Habit

Habit was frequently mentioned by participants, both with regard to their use of technology and related to participating in physical activity. Habit was also linked by many Veterans to their previous military service. Our interview guides did not specifically probe participants about habit, making the prominent emergence of habit notable. In the discussion of habit, some participants described how memory and learning contribute to the development of habitual use of technology.

Memory appears to play a dual role in use of the technology—in remembering to use the technology and how to use the technology.

Something to remind me. But, I’m going to have to set a schedule of when I actually do this.

P13

It’s a problem with my memory. The program to me seems fine if I can remember how to go through it.

P15

Learning was discussed often as a period of trial and error where users would become more facile with using the app with greater experience.

Once I learned this app and spent just a little bit of time with it, I’ll be good with it. I don’t see any problem with it.

P23

Ultimately, these efforts are expected to result in habitual use of VA FitHeart.

If I were to [use the app] religiously, every day do it, then it’d be force of habit.

P08

### Hedonic Motivation

Most comments about pleasure derived from using technology were general in nature. Comments about VA FitHeart itself were less strongly pleasurable in nature, but generally positive.

But I like the looks of the app and I like what it’s set up to do.

P28

### Price Value

Though participants mentioned price and cost related to other technologies and mobile apps, price value was infrequently mentioned linked to our mobile app, which will be free for general use.

I think in the end, you could save people, or patients, money.

P35

### Physical Activity

In our interviews, we identified many of the common barriers and facilitators to physical activity and participation in CR that have been described in previous studies (Multimedia Appendix 5) [34].

Attitudes expressed included general attitudes toward physical activity, as well as comments related to health benefits and the influence of other medical conditions. Many participants commented on subjective norms including the influence of pets, family, and health care providers. Participants frequently mentioned themes relating to perceived behavior control such as goals, habit, motivation, work (as either a facilitator or barrier), and travel or transportation.

We identified one notable emergent theme that does not clearly fall within a single TPB construct and that has not been well described before: the role of military service in physical activity.

### Military Service

Though we specified a priori categories, the topic of military service was mentioned so frequently by our population that we created an emergent category for military service, which may be uniquely important to our patient population. In our population of US military Veterans, almost all Veterans reported their time of military service as a physically active time in life. Their time in military service was often central to their experience related to physical activity.

When I joined the service I was very fit. I usually did physical activity in the morning and sometimes in the afternoon also, an average of 2.5 hours a day, 4 to 5 days a week.

P7

Additionally, many Veterans described their time after discharge as a particularly inactive time.

I hadn’t worked out since the military. It had been like 18 years since I’d set foot in a gym.

P45

## Discussion

### Principal Findings

We found that iteratively revising a mobile app for CR based on user feedback resulted in significant improvements in the usability of the mobile app. Using a theory-based approach, we revealed interest in using a mobile app to track physical activity and health measures and to share data with providers. Patients expected to have training on how to use the mobile app. On the basis of participant comments, establishing habit, both with regard to physical activity and mobile app use, is anticipated to be a key contributor to adoption of this technology.

This is the first theory-based investigation of the usability of a mobile app for CR. It is known that interventions based on theory are more likely to be effective [20]. Other technology-facilitated interventions for CR have been studied, with promising results [18,35,36]. However, these studies did not describe theoretical considerations related to health behaviors or technology use, so we know little about how the interventions influenced patient behavior to achieve their results. Other investigators have also reported the development of theory-based mobile CR platforms, but results of their use and efficacy have not been reported [37]. Having a framework for understanding how an intervention produces its effects will be important for studying its impact and adapting interventions beyond research studies. We found that constructs from UTAUT2 [28], especially performance expectancy, effort expectancy, social influence, facilitating conditions, and habit appear to play an important role in use of mobile technology for CR.

Patients in our study desired the ability to track physical activity and health measures with an easy-to-use mobile app, confirming findings from previous studies [18,38,39]. Though some participants expressed a desire for additional features to the mobile app, such as integration with device or peripheral sensors for motion or location, it was commented that these features were not essential. In general, VA FitHeart received praise for its simplicity.

It has previously been reported that people have little desire to share their personal fitness data with their providers [40]. We found that many patients expected to share their data with their health care providers and viewed this as a key advantage to using VA FitHeart. It may be that apps designed to be used for health conditions are viewed differently than consumer personal fitness trackers. Other studies of patient-provider digital communication interventions have demonstrated high levels of satisfaction [41]. Theory related to physical activity behavior and technology use behavior would suggest that sharing data with providers has the potential to influence patient use of a mobile app to promote physical activity through subjective norms and social influence [24,28], and our finding that patients expect to share their data with providers is consistent with this.

Many participants expressed an expectation for in-person training on use of the mobile app, in addition to on-demand help online, via telephone, or from family and friends. Previous studies of older adults have also revealed a preference for in-person training and the influence of family and friends [42,43]. It has also been suggested that technology training for older adults may need to be geared toward their needs and learning styles [44,45]. As older adults are less likely to use mobile technology than younger adults, interventions and training geared toward older adults may be necessary [46]. Together, this suggests that interventions for technology-facilitated CR should include opportunities for in-person training of participants on use of the technology, in addition to on-demand help.

Habit was frequently and prominently mentioned by Veterans as a factor that will be important, both for using the mobile app and participating in physical activity. UTAUT2 describes experience and habit as related concepts, with experience being necessary but not sufficient for establishment of habit [28]. In our study, patients frequently discussed memory and learning as prerequisites to habitual use, rather than mentioning experience. For our older population, experience may need to be considered more broadly with regard to repetition and retention of learned behaviors to establish habitual use. In addition, mention of habit was linked by some Veterans to their military service, and it is possible that experience in military service influences how habit is developed in our population. Interestingly, with iterative revisions to improve the usability of the mobile app, we noticed improvements in participant ratings of expected habit and hedonic motivation with use. Though effort expectancy is not theorized to influence habit or hedonic motivation [28], it may be that the usability of a mobile app influences expected adoption of regular use and pleasure derived from the mobile app. Other studies have found that for new users of online fitness communities, self-regulatory motives influence habitual use but that for experienced users, social motives and enjoyment play a larger role [47]. It has also been observed that for social apps, perceived usefulness and hedonic motivation influence habit, which may mediate the effects of perceived usefulness and hedonic motivation on technology use behavior [48]. Together, this suggests that mobile apps that are easier to use may be both more enjoyable to use and more likely to be perceived as habit-forming.

### Limitations

Several limitations to our findings should be considered. We had a small sample size of Veterans and only one female, so our population may not fully represent the population or non-Veteran populations. As not all eligible patients agreed to participate, our findings may not be representative of the entire eligible population. Due to our small sample size, we may not have truly achieved thematic saturation of all factors associated with the use of mobile technology for CR. However, our sample did provide valuable feedback that resulted in improved usability. Additionally, we studied VA FitHeart in a usability testing environment and not in a real-world environment. Further testing is needed in a real-world environment to determine whether other factors are important to use.

### Conclusions

With participant feedback and iterative revisions, we significantly improved the usability of a mobile app for CR. Patient expectations for using a mobile app for CR include tracking health metrics, introductory training, and sharing data with providers. Iterative theory-based mixed-method evaluation may be useful for improving the usability of health technology.

## Appendix

Multimedia Appendix 1 Mobile Application Use questionnaire.

Multimedia Appendix 2 Interview guides.

Multimedia Appendix 3 Screening and enrollment of participants.

Multimedia Appendix 4 Quotations related to concepts from the extended unified theory of acceptance and use of technology.

Multimedia Appendix 5 Quotations related to concepts from the Theory of Planned Behavior.

## Abbreviations

CR: cardiac rehabilitation
SUS: System Usability Scale
TPB: Theory of Planned Behavior
UTAUT: Unified Theory of Acceptance and Use of Technology
UTAUT2: Unified Theory of Acceptance and Use of Technology extension for consumer use of technology
VA: Veterans Affairs
