# Supplementary material for: VA FitHeart, a Mobile App for Cardiac Rehabilitation: Usability Study
Source: JMIR Hum Factors. 2018 Jan 15;5(1):e3. doi: 10.2196/humanfactors.8017 (PMC5789161; doi:10.2196/humanfactors.8017)
Supplement: Multimedia Appendix 1 [file humanfactors_v5i1e3_app1.pdf]

Appendix 1. Mobile Application Use Questionnaire

Mobile Application Use Questionnaire	
**Question**	**Factors**
I find using the mobile application useful in my daily life.	Performance Expectancy
Using the mobile application increases my chances of achieving things that are important to me	Performance Expectancy
Using the mobile application helps me accomplish things more quickly	Performance Expectancy
People who are important to me think that I should use the mobile application	Social Influence
People who influence my behavior think that I should use the mobile application	Social Influence
People whose opinions that I value prefer that I use the mobile application	Social Influence
I have the resources necessary to use the mobile application	Facilitating Conditions
I have the knowledge necessary to use the mobile application	Facilitating Conditions
The mobile application is compatible with other technologies I use	Facilitating Conditions
I can get help from others when I have difficulties using the mobile application	Facilitating Conditions
Using the mobile application is enjoyable	Hedonic Motivation
The use of the mobile application has become a habit for me	Habit
I intend to continue using the mobile application in the future	Behavioral Intention
I will always try to use the mobile application in my daily life	Behavioral Intention
I plan to continue to use the mobile application frequently	Behavioral Intention
