# Supplementary material for: VA FitHeart, a Mobile App for Cardiac Rehabilitation: Usability Study
Source: JMIR Hum Factors. 2018 Jan 15;5(1):e3. doi: 10.2196/humanfactors.8017 (PMC5789161; doi:10.2196/humanfactors.8017)
Supplement: Multimedia Appendix 2 [file humanfactors_v5i1e3_app2.pdf]

Appendix 2. Interview Guides

Mobile Cardiac Rehabilitation Interview Guide

[Generic prompts: If responses are limited or require clarification, probes may be used to elicit more detailed responses. Probes should use words or phrases presented by the participant using one of the following formats:
1. What do you mean by _____________ ?
2. Tell me more about _____________ ?
3. Give me an example of _____________ ?
4. Tell me about a time when _____________ ?]

1. Describe your experience using mobile devices, such as a phone or a tablet?

   Specific Probes [if needed]

   a. Describe your experience using mobile applications?
   b. Describe your experience using mobile applications for fitness or health?
   c. Give me an example of a mobile app that you use often?
   d. Can you give me an example of a mobile application that you downloaded, but don't use?

2. What role does physical activity play in your life?

   Specific Probes [if needed]

   a. Tell me about a time when you were physically active on a regular basis?
   b. Tell me about a time when you were less physically active?
   c. What role, if any, does technology have in you being physically active?

3. What do you know about cardiac rehabilitation?

   a. *If the participant indicates they have previously participated in cardiac rehab:* Tell me more about your experience with cardiac rehabilitation.

      Specific Probes [if needed]

      i. What was helpful about the experience?
      ii. What was hard about the experience?

   b. *If the participant doesn't know anything or has limited knowledge, explain:*

Cardiac rehabilitation is a program to help improve heart health in people like you. It involves exercise training and physical activity and helps to teach you how to manage your heart disease. People usually participate in about 3 sessions a week for about 3 months.

4. You are eligible for cardiac rehabilitation. What are your plans, if any, for participating in cardiac rehabilitation?
    a. *If no plans:* What would get you to participate?

Do you have any questions for us?

Is there anything else you would like to add?

Usability Interview Guide

[Generic prompts: If responses are limited or require clarification, probes may be used to elicit more detailed responses. Probes should use words or phrases presented by the participant using one of the following formats:
1. What do you mean by _____________ ?
2. Tell me more about _____________ ?
3. Give me an example of _____________ ?
4. Tell me about a time when _____________ ?]

1. Tell me about your experience using the mobile app today.
    a. Did you have trouble with any of the features?
    b. [if needed] I noticed that the ______ feature took you longer than some of the others. Tell me more about that?
2. Would you use this mobile application?
    a. If yes, How would you use this mobile application? *Specific probes [if needed]*
        i. Is there anything that would help you use the mobile application?
        ii. Is there anything that would keep you from using the mobile application?
    b. If no, What would keep you from using the mobile application?
3. What can we do to make this mobile application better?

    Specific probes [if needed]

    a. What features should we change?
    b. Are there other features we should add?

Do you have any questions for us?

Is there anything else you would like to add?
