# Supplementary figures and images for: VA FitHeart, a Mobile App for Cardiac Rehabilitation: Usability Study
Source: JMIR Hum Factors. 2018 Jan 15;5(1):e3. doi: 10.2196/humanfactors.8017 (PMC5789161; doi:10.2196/humanfactors.8017)

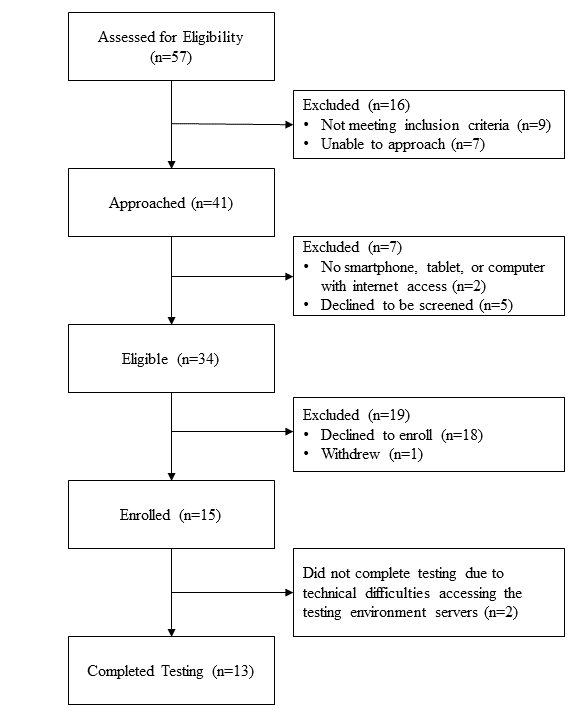

Supplement: Multimedia Appendix 3 [file humanfactors_v5i1e3_app3.png]
