# Supplementary material for: VA FitHeart, a Mobile App for Cardiac Rehabilitation: Usability Study
Source: JMIR Hum Factors. 2018 Jan 15;5(1):e3. doi: 10.2196/humanfactors.8017 (PMC5789161; doi:10.2196/humanfactors.8017)
Supplement: Multimedia Appendix 4 [file humanfactors_v5i1e3_app4.pdf]

Multimedia Appendix 4. Quotations related to concepts from the extended unified theory of acceptance and use of technology.

Theme		Quotation
**Performance expectancy: the degree to which using a technology will provide benefits in performing certain activities**		
	General	*As long as it's working as intended, I would definitely use it. [P28]*<br>*I think it's going to encourage me to actually take some positive steps, exercising on a regular basis. [P07]*<br>*It's much easier to keep it on a phone, it's instantaneous and a lot more accurate, I think personally. There's less chance of losing it. [P23]*<br>*But I think that the idea of an app that records all of the information that this app is doing will be very valuable. Actually somewhat of a motivation for me to do this thing. [P28]*
	Setting goals	*The fact that it does save these goals for you and then you'll be able to compare those eventually with what you're actually doing as far as the exercise goals. [P23]*<br>*I just like participating with it [the app], to see if I can reach the goal. [P11]*<br>*For me, that's the only thing I felt like I struggled with. Setting a realistic goal at the beginning. [P47]*<br>*It's important to set goals like that, that are attainable. [P23]*
	Tracking health measures	*I think it'll be good to track my exercise and to see what I'm doing. Because there's information that I don't usually track, such as, I think it was the number of steps. I can*

		probably do a better job of tracking that way. [P45]<br>*The simplicity of the application works perfectly with what I'm doing with rehab. It has everything I do today. I have to do a weight check, and I can put my weight in. I can put my Blood pressure, my weight, my height, my glucose level. This keeps tabs on everything I currently need to do and gives me the secure ability to know that I have structure, that I can follow the structure and if I get away from the structure I can go back and see if I did it right. [P07]*<br>*Once I have an exercise program, I could see the value in it. To track my progress. To keep me on track. So that I know I got this many minutes do this week, that I'd have to buckle down and get it done. [P44]*
	Other features	*I like that it's simple and that it has not only components for exercise but that you can look up or research diet and quitting smoking and calories and all of that too. [P45]*<br>*But memorizing, writing it down and then getting it into your computer, if that was all done while you're doing activities and stuff that would be a big help. But if they can't, this is still a good app. Still helpful. [P28]*
**Effort expectancy: the degree of ease associated with use of technology**		
	Easy to use	*I found it very easy to use. [P23]*<br>*The flow is easy, I don't see any kind of connectivity difficulty and anything that's*

		basic information, unless the user doesn't know anything about how to connect to the keyboard or anything like that, that was the only problem I had. [P07]
	Simple	*It was pretty easy...I like that it's simple. [P45]*<br>*The flow is very simple. [P07]*<br>*It doesn't seem cumbersome. It seems pretty straight forward. [P13]*<br>*It's pretty cut and dry. Like I said, if you have this menu thing up there, you can't go wrong. If I get stuck, I can always just go back to menu. [P45]*
	Vision	*The only downside I see for me is with my vision; the fonts are a little small. I would definitely need to use my reading glasses to read it. [P44]*<br>*If it was on a bigger screen that would help too. I could read it easier. [P15]*
	Prominence	*The virtual keypad. The problem I have right now is that it's hard to find. [P07]*<br>*One of the things I liked about the graph is it's bold, it's big. [P07]*<br>*The settings to change your goals are very easy to reach and very prominent. [P23]*
	Barriers to use	*It wasn't as intuitively what I'm used to with some of the other things. [P19]*<br>*It was frustrating. Like I said, it wasn't as it should be. It should be more where one would lead into the other, a simple route. [P19]*
	Passwords and codes	*My memory is a little down on trying to remember. They want you to have a different code with different banks. I have a couple of different banks and you have to have a different code to get into them, and*

		that confused me. [P35]<br>*Once it's in my computer at home, I don't need to remember the password and do the procedure again. And so I don't remember the password. I've gotten in the habit now where I've got it in my wallet on my card where I have various passwords and name IDs for each system. [P19]*
	Familiarity	*It's just a matter of familiarity...if I had time to just play with the app ahead of time, I would learn where everything was and then I would know that's where I should be looking for it. [P28]*<br>*I'm not used to this. Once I get used to it, I'll know where everything is. [P40]*
**Social influence: the extent to which users perceive that important others believe they should use a particular technology**		
	Sharing data with providers	*That way they [providers] can see what's been going on, without me telling them. [P40]*<br>*I like the fact that I can put all of that and track it, and that my doctors can as well. I can show my doctor what I've been working on. [P45]*<br>*…the security that the doctors and nurses and whoever cares for that person, has access to the information and can see what he sees in a quick second. So he doesn't have to go to see the doctor and ask if he saw his heart rate or blood pressure. [P07]*
	Provider communication	*I didn't see it here, is if there's an ability for the app to connect to the team nurse. [P07]*
	Provider	*I started using [a fitness app] because my*

	recommendations	*doctor wanted to know the distance and what my heart rate was. [P31]*
	Family or peer influence	*My wife is my second memory. If I went for a walk she'd tell me to get my little computer out and to put down what I did. [P53]*
	Social networking	*And, I like the [link] where I can look up people that have the same issues. [P55]*<br>*They have online support, but I don't use that stuff, so. And I went to meetings twice, and it just wasn't for me. I prefer to just do it on my own with the app. [P54]*
**Facilitating conditions: perceptions of the resources and support available to perform a behavior**		
	Training	*Initially, training with a technical person, instead of me relying on myself. [P8]*<br>*If I could be taught how to do it, yeah, I'd like to. Especially if it could help with this. I'd like that. [P19]*<br>Interviewer: *But you're thinking that you would want someone to sit down with you.*<br>Participant: *In the beginning, absolutely. A little while to show them the app and everything and get them on the right trail, absolutely. [P35]*<br>*Come in for some classes or whatever and have progress doing it. Maybe just a little bit, one phase at a time. [P19]*
	On-demand help via phone or in person	*I would need a phone number to call. Or if the website provided a link to ask. But I'd just as soon a phone call probably. [P8]*<br>*…if I had problems I'd try to find out how to fix it on this or call you. [P40]*
	Family or peer assistance	*But I've got 3 boys that are all pretty much wizards at it, but I'm not. I'm sure I can*

		learn it or if they punch in the application so that it could come right up, I'd be fine. [P19]<br>*I have her [wife] do everything for me when it comes to that [using a smartphone]. [P15]*<br>*If I went for a walk she'd [wife] tell me to get my little computer out and to put down what I did. [P44]*
**Habit: the extent to people tend to perform behaviors automatically because of learning**		
	Memory	*I have memory issues as well, it's other things. Remembering to do it is one of my things. [P44]*<br>*Something to remind me. But, I'm going to have to set a schedule of when I actually do this. [P13]*<br>*It's a problem with my memory. The program to me seems fine if I can remember how to go through it. [P15]*
	Learning	*And the other thing, because it's a new app, there's a little bit of a learning curve, I need to learn where to look for specific things and to be able to navigate a little better. [P28]*<br>*Once I learned this app and spent just a little bit of time with it, I'll be good with it. I don't see any problem with it. [P23]*
	Habit	*Make myself do this on a very consistent basis. It's going to have to become a habit. [P13]*<br>*If I were to [use the app] religiously, every day do it, then it'd be force of habit. [P8]*<br>*It'll be important for me to keep using it frequently. Otherwise I may have some*

		difficulty if I don't. At my age, learning is memory, and I don't have a problem with the memory so long as I use it all of the time. If I don't use it frequently, then I may need help. [P8]
**Hedonic motivation: the fun or pleasure derived from using a technology**		
	General	*If I wanted to do more, there's a link. So that's a positive aspect where the user doesn't have to wait and if he wants to continue to learn more about the information. [P7]*<br>*I use my phone. Basically if I get personal emails I use it. My phone is more for that use and also for quick identification and using it with Facebook. Facebook, email and just search requests. [P7]*<br>*Reading, using word puzzles, crossword puzzles. Help to keep your mind moving, so you don't lose as much. [P15]*<br>*A lot of stuff. Information. Learning. Answering emails. Just investigating things. Being I'm retired I have a lot of time to sort of look things up. It's in my nature to be inquisitive. [P8]*
	App-specific	*As a person who is very familiar and comfortable with technology, I would personally use it a lot. [P23]*<br>*But I like the looks of the app and I like what it's set up to do. [P28]*<br>*It can show me if I'm exercising too much or not exercising enough. If I'm given limits that would work out well, if I knew what the limits were. And if I needed to more because I'm not doing enough, not*

		knowing my body as well as I should, I thought I knew it pretty well, but I think with all of this information it could be a lot better for me. [P35]
**Price value: the tradeoff between the perceived benefits of the app and the monetary cost for using it**		
	Cost-benefit expectations	*I think in the end, you could save people, or patients, money. [P35]*<br>*I’ve downloaded a couple apps, but there’s not much to download even. So yeah. I just don’t buy a lot of that stuff because I think it’s just junk. [P40]*
