# Supplementary material for: VA FitHeart, a Mobile App for Cardiac Rehabilitation: Usability Study
Source: JMIR Hum Factors. 2018 Jan 15;5(1):e3. doi: 10.2196/humanfactors.8017 (PMC5789161; doi:10.2196/humanfactors.8017)
Supplement: Multimedia Appendix 5 [file humanfactors_v5i1e3_app5.pdf]

Multimedia Appendix 5. Quotations related to concepts from the Theory of Planned Behavior.

Concept		Quotation
**Attitudes—physical activity: beliefs about the behavior and evaluation of outcomes resulting from the behavior**		
	General	Interviewer: *But are there certain things that make it easier for you to be physically active?*<br>Participant: *I just like doing it. [P11]*<br>*Besides knowing that's healthy for me, it's also something I enjoy. [P8]*
	Other medical conditions	*When you get my age, you'll feel them bones cracking. [P16]*<br>*…until I had my stroke, uh, there was no problem whatsoever. I would go to all the classes. And then I'd either run or walk daily. [P11]*<br>*I mainly just walk in the pool. Because my hips are getting bad. [P40]*
	Desire for better health	*I only walk. It's pretty much a habit. I know it's healthy for me, and I enjoy it for the most part. [P8]*<br>*I sort of feel, with my recent history, that I've done it and am working hard to keep myself healthy. And I like that. [P19]*<br>*I would like to improve, if possible, the breathing and my heart function. [P13]*<br>*I feel like my job is to stay as healthy as possible and exercise. [P45]*
	Guilt	*I feel guilty if I don't do it. Good or bad. There's definitely times I get on that thing and think Ah, there's no way I can go twenty minutes. I just sort of I gotta do it, and I guess the endorphins kick in maybe*

		10 minutes later. [P8]
**Subjective norms: beliefs about others' opinions of the behavior and their influence on motivation**		
	Pets	*I walk with my dog and we go almost 30 blocks. [P35]*<br>*She [pet dog] loves to go down to the...We go down to the river. When it goes along the Green River. The Green River Trail. It interacts with the Interurban trail in the valley. Goes from Seattle all the way down to, where does that thing end, man it ends way down there, almost to Sumner. It doesn't go to Sumner, but Pacific or somewhere in there. I mean. I just, We just walk the park. There's this little park down [inaudible] called Foster Park and it's got a circle you can walk around. We can do that in ten to fifteen minutes. Sometimes I get on the interurban trail and go for forty-five minutes to an hour, and then I end up paying for that. [P16]*
	Grief or family obligations	*My wife was diagnosed with cancer and dying from it, and I just lost any incentive I had. I didn't care if I rehabilitated my heart or not. [P28]*<br>*And she's got a developmentally disabled daughter that I'm sort of watching over while she's gone too. That's got to take priority over any of this other stuff. [P19]*<br>*I had about a 3 year period where I was taking care of my first wife. When she was fighting cancer. She wanted me to be there with her, and that's what I did. [P13]*
	Provider influence	*If the VA wants me to, I'll do it. I think I get enough exercise and do enough activities on my own that I don't need it,*

		but it's not my decision, it depends on how my health holds up and how the VA thinks my health is holding up. [P15]<br>*…biggest thing out of it for me was the encouragement. [P7]*<br>*…he helped me to see the improvements that would be made if I continued on the path that I was on. [P45]*
**Perceived behavioral control: personal agency for performing the behavior**		
	Goals	*If you don't have goals there's really no motivation. [P28]*<br>*So, I was interested after I got my stents put in, to see if I could get back up to that level. Currently, my goal is to run in the [name of race] which is next month in May. So it's important to set goals like that, that are attainable. [P23]*
	Habit	*It's become a habit with me, for better or worse. [P08]*<br>*I just try to replicate it every day. Every day it's twenty minutes. I'm sweaty when I'm done with it. [P08]*<br>*But I do have a structure that I have to do some kind of cardio, even if it's a stationary bike or sitting down exercise. I try to have some kind of physical activity done every day. [P07]*<br>*I'm very consistent. [P19]*<br>*I think the whole thing is just to get people used to doing something again, and hopefully give you the bug, so you can take it and do it in your own neighborhood. [P45]*
	Motivation	*I have a motivation problem with doing the exercising. I'm not really motivated. [P28]*

		Inherently, I'm lazy. So if there's something that could keep me motivated. [P13]
	Work	Facilitator<br>*My work demanded high, high-end physicality. [P16]*<br>*I work right now, 6 days a week. I walk 4 or 5 hours every night on my job. [P13]*<br>Barrier<br>*The jobs I had there were rather sedentary for the most part. [P28]*<br>*When I worked full time, I honestly was just too exhausted to ever want to work out or even walk after work. [P45]*<br>*I was going to the gym and then my job hours and everything changed to make it relatively difficult if not impossible. [P13]*<br>*It [cardiac rehab] would have to be that it doesn't interfere with my work. [P23]*
	Travel or transportation	*It's too much of a drive. [P28]*<br>*There's no place close to me because of where we live. [P15]*
**Emergent theme—military service**		
	Military service	*When I joined the service I was very fit. I usually did physical activity in the morning and sometimes in the afternoon also, an average of 2.5 hours a day, 4 to 5 days a week. The majority was based on cardio based running, speed walking and some kind of sports activity. I went gradually down after I left the service and started going back up after I had my stroke. I started going back up to moderate to vigorous activity afterwards. [P7]*<br>*In the military I was obviously in top physical condition. [P45]*

		When I got back from Vietnam I wasn't doing anything. [P11]<br>*I basically started slowing down, I felt it wasn't really needed, I just wanted to stay fit, but that turned into sluggishness and laziness and then I stopped about a year or a year and a half after I left the service, I just basically semi-stopped. [P7]*<br>*I hadn't worked out since the military. It had been like 18 years since I'd set foot in a gym. [P45]*<br>*A lot of these men, you know, had been out of the military for 35 years, and may not have never been in a gym since then. [P45]*
